# COPIOUS: A gold standard corpus of named entities towards extracting species occurrence from biodiversity literature

**DOI:** 10.3897/BDJ.7.e29626

**Published:** 2019-01-22

**Authors:** Nhung T.H. Nguyen, Roselyn S. Gabud, Sophia Ananiadou

**Affiliations:** 1 The National Centre for Text Mining, University of Manchester, Manchester, United Kingdom The National Centre for Text Mining, University of Manchester Manchester United Kingdom; 2 University of the Philippines Diliman, Quezon City, Philippines University of the Philippines Diliman Quezon City Philippines; 3 University of the Philippines Los Baños, Los Baños, Philippines University of the Philippines Los Baños Los Baños Philippines

**Keywords:** Biodiversity, text mining, named entity recognition, species occurrence, gold standard

## Abstract

**Background**

Species occurrence records are very important in the biodiversity domain. While several available corpora contain only annotations of species names or habitats and geographical locations, there is no consolidated corpus that covers all types of entities necessary for extracting species occurrence from biodiversity literature. In order to alleviate this issue, we have constructed the COPIOUS corpus—a gold standard corpus that covers a wide range of biodiversity entities.

**Results**

Two annotators manually annotated the corpus with five categories of entities, i.e. taxon names, geographical locations, habitats, temporal expressions and person names. The overall inter-annotator agreement on 200 doubly-annotated documents is approximately 81.86% F-score. Amongst the five categories, the agreement on habitat entities was the lowest, indicating that this type of entity is complex. The COPIOUS corpus consists of 668 documents downloaded from the Biodiversity Heritage Library with over 26K sentences and more than 28K entities. Named entity recognisers trained on the corpus could achieve an F-score of 74.58%. Moreover, in recognising taxon names, our model performed better than two available tools in the biodiversity domain, namely the SPECIES tagger and the Global Name Recognition and Discovery. More than 1,600 binary relations of Taxon-Habitat, Taxon-Person, Taxon-Geographical locations and Taxon-Temporal expressions were identified by applying a pattern-based relation extraction system to the gold standard. Based on the extracted relations, we can produce a knowledge repository of species occurrences.

**Conclusion**

The paper describes in detail the construction of a gold standard named entity corpus for the biodiversity domain. An investigation of the performance of named entity recognition (NER) tools trained on the gold standard revealed that the corpus is sufficiently reliable and sizeable for both training and evaluation purposes. The corpus can be further used for relation extraction to locate species occurrences in literature—a useful task for monitoring species distribution and preserving the biodiversity.

## Introduction

### Background

Biodiversity plays a central role in our daily lives, given its implications on ecological resilience, food security, species and subspecies endangerment and natural sustainability. Research in this domain has recently seen accelerated growth, leading to the "big data" scenario of the biodiversity literature. For instance, the Biodiversity Heritage Library (BHL)*^1^—a consortium of natural history and botanical libraries, currently holds over 55 million digitised pages of legacy biology text from the 15th-21st centuries, representing a huge amount of textual content (Gwinn and Rinaldo 2009). Applying text mining tools to convert the content into a machine-readable form and enable data-driven discovery is important to biodiversity science (Thessen et al. 2012).

Text mining can be defined as a process that aims to extract interesting and non-trivial patterns or knowledge from unstructured textual data in document collections (Ananiadou and McNaught 2005, Feldman and Sanger 2007). Text mining has successfully been applied to the biomedical literature (Arighi et al. 2013, Wei et al. 2013, Mihăilă et al. 2015, Ananiadou and Thompson 2017) and more recently, it has also been employed in the biodiversity domain to unlock knowledge hidden in the literature (Ulate 2014, Barrios et al. 2015, Batista-Navarro et al. 2016, Batista-Navarro et al. 2017, Parr and Thessen 2018, Chaix et al. 2018).

This work is part of the COPIOUS project*^2^, which aims to produce a knowledge repository of Philippine biodiversity by applying text mining-based big data analytics to biodiversity literature. The repository will be a synergy of different types of information, e.g. taxonomic and species occurrence, thus providing users with a comprehensive view on species of interest that will allow them to carry out predictive analysis on species distributions. To this end, the repository needs to include several types of information, such as taxons or species names, habitats, geographical locations, persons and temporal expressions. We therefore need to build text mining tools that can detect such information from biodiversity text.

Most text mining work in the biodiversity domain has focused on discovering species names; tools designed for this purpose include TaxonGrab (Koning et al. 2005), TaxonFinder (Leary et al. 2007), OrganismTagger (Naderi et al. 2011), NetiNeti (Akella et al. 2012), SPECIES tagger (Pafilis et al. 2013), BiOnym (Berghe et al. 2015) and Global Names recognition and discovery tool (GNRD) (Pyle 2016). Along with these tools are two corpora, i.e. Linnaeus (Gerner et al. 2010) and S800 (Pafilis et al. 2013), which consist of annotated scientific names and vernacular names of species. The tools and two corpora for species names recognition are not, however, sufficient for our work, because we need to identify additional categories of entities. Unlike Linnaeus and S800, other corpora are annotated with multiple entity categories, e.g. Bacteria Biotope (Delėger et al. 2016), ACE 2005 (Walker et al. 2006), CoNLL 2003 (Kim Sang and Meulder 2003) and MUC-7 (Chinchor 2001). Bacteria Biotope is a corpus focused on microorganisms, i.e. bacteria and their habitats. The corpus includes annotations of bacterial taxon names and habitats, which are related to our own requirements. However, in the COPIOUS project, to support the conservation of the Philippine biodiversity, our initial emphasis is on organisms that are highly endangered with extinction, such as birds, fish, mammals and plants; microorganisms will be dealt with in future work. As a result, the types of taxon names and habitats annotated in Bacteria Biotope and recognised by tools trained on the corpus (Björne and Salakoski 2015, Karadeniz and Özgür 2015, Lavergne et al. 2015) are not suitable for supporting our immediate aims. While Bacteria Biotope concerns the biomedical domain, the other corpora mentioned above, i.e. ACE 2005, CoNLL 2003 and MUC-7, belong to the general domain, e.g. newswire, weblogs, broadcast news etc. Both ACE 2005 and CoNLL 2003 include annotations of persons and locations; MUC-7 is annotated with persons, locations and temporal expressions. However, since the two corpora do not include text from the biodiversity domain and since their annotations do not match with our target of species occurrence, we do not make use of them, or tools developed using them, in this work. Differences between these corpora and our own corpus will be detailed in the following section.

Up until now, there are no existing resources (either corpora or tools) that correspond directly to our area of interest. To address this situation, we have constructed the COPIOUS corpus— a gold standard corpus annotated with five different categories of entities that are relevant to biodiversity: Taxon, Geographical location, Habitat, Person and Temporal expression. The basis for the gold standard corpus was a set of English documents downloaded from the Biodiversity Heritage Library (BHL). We randomly selected 668 documents and asked our annotators to manually mark up the documents based on our guidelines. The average inter-annotator agreement of 78.22% F-score demonstrated that the annotations in our corpus were consistent and reliable.

To demonstrate the utility of the gold standard corpus, we used it to assist in the development of two types of text mining tools necessary for the construction of a biodiversity knowledge repository, i.e. named entity recognition (NER) and relation extraction. We trained two NER tools on the gold standard using two different machine learning approaches, i.e. Conditional Random Fields (CRF) (Lafferty et al. 2001) and Bi-directional Long Short Term Memory (BiLSTM) (Espinosa et al. 2016), which constitute state-of-the-art models in statistical and deep learning methods, respectively. We achieved similarly high levels of performance for NER using both methods, with the best performance of 74.58% F-score being achieved by the BiLSTM model. In comparison to other automatic species name recognisers, i.e. SPECIES tagger (Pafilis et al. 2013) and GNRD (Pyle 2016), the tool trained on our gold standard produced superior performance.

For the relation extraction experiment, we aimed to extract relations that can be used to form species occurrence records. These relations include Taxon-Geographical location, Taxon-Habitat, Taxon-Person and Taxon-Temporal expression. Since we do not have any gold standard annotations for these relations, we applied PASMED (Nguyen et al. 2015), an unsupervised relation extraction system for the biomedical domain, on top of the gold standard entities. The resulting relations can be used to augment primary species occurrence data such as the Global Biodiversity Information Facility (GBIF)*^3^ in a semi-automatic manner.

### Related work

There are two corpora that are annotated with taxon entities similar to our work, i.e. Linnaneus (Gerner et al. 2010) and S800 (Pafilis et al. 2013). Linnaeus (Gerner et al. 2010) consists of 100 full papers randomly selected from the PMC open access set. All mentions of species in the text were manually annotated and normalised to the NCBI Taxonomy database*^4^. A total of 72% of these mentions are common names, including those that do not directly convey species names such as "participant", "patient", "child" and "boy". In contrast to Linnaeus, S800 (Pafilis et al. 2013) was constructed using 800 PubMed abstracts. In order to maximise the diversity of species names in the corpus, these abstracts were selected from eight categories based on their journal scopes: bacteriology, botany, entomology, medicine, mycology, protistology, virology and zoology. Table 1 illustrates the diversity of S800; the size of S800 is much smaller than that of Linnaeus, but the number of species mentions annotated is only slightly less than Linnaeus. An analysis of S800 revealed that a number of mentions are strain names, e.g. "R-40509(T)", "M2T2B15" and "*Cryptococcus neoformans* JEC21", which do not align well with our design goals for the biodiversity domain.

Although Linnaeus and S800 are useful corpora and are large enough to allow training of a machine learning-based NER, they were developed for the biomedical domain rather than the biodiversity domain. Additionally, most of the annotated scientific names in both corpora are in the format of binomial nomenclature, i.e. names with two parts of genus and species, which overlooks other variants of scientific names, e.g. family names, genus names and names including information pertaining to authority. We therefore decided to construct a novel gold standard corpus for biodiversity species names, whose annotations cover both variants of scientific names and vernacular names.

Previous work on recognising taxonomic names has mostly used dictionary-based approaches, i.e. text is matched against a predefined dictionary of species names. TaxonGrab (Koning et al. 2005) is an NER tool that can identify organism scientific names from existing documents. TaxonGrab was implemented by combining taxonomic nomenclature rules, a lexicon of English words extracted from WordNet (Miller 1995) and the SPECIALIST lexicon (McCray et al. 1994). TaxonFinder (Leary et al. 2007) is another tool to recognise scientific names at all taxonomic ranks, e.g. species, genera and subspecies, using a dictionary-based approach. Linnaneus (Gerner et al. 2010), OrganismTagger (Naderi et al. 2011) and the SPECIES tagger (Pafilis et al. 2013) also used dictionary-based approaches, but they have the important feature of being able to recognise vernacular names in text in addition to scientific ones.

BiOnym (Berghe et al. 2015) is another scientific name-matching system that implements a sequence of matchers, e.g. trigram matcher, Levenshtein matcher and fuzzy matcher. Unlike the previously described tools that can detect fixed names, BiOnym allows users to select their preferred list of species names to be incorporated into the system. Global Names Recognition and Discovery (GNRD) is an online service of the Global Names Architecture (Pyle 2016), which can find scientific names of species on web pages, documents and free-form texts. GNRD is a combination of TaxonFinder (Leary et al. 2007) and NetiNeti (Akella et al. 2012).

NetiNeti (Akella et al. 2012) is a machine learning-based tool that can discover scientific names of species from biomedical and biodiversity texts. NetiNeti firstly generates candidate names using rules for scientific names and then applies Naive Bayes and Maximum Entropy to classify the candidates. It should be noted that the authors did not use an annotated corpus to train NetiNeti, but rather, they automatically generated positive and negative samples based on a list of 5,000 species names.

In addition to species names, extracting locations and habitats of species from literature is also important for domain experts, because such information can help to answer questions such as "What lives here?" or "What is the distribution of this organism?" (Parr and Thessen 2018). Corpora annotated with locations and/or habitats include Bacteria Biotope (Delėger et al. 2016), ACE 2005 (Walker et al. 2006), CoNLL 2003 (Kim Sang and Meulder 2003) and MUC-7 (Chinchor 2001).

Bacteria Biotope (Delėger et al. 2016) consists of 161 PubMed abstracts annotated with bacterial taxon names and habitat mentions. The selected abstracts were firstly pre-annotated with the entities of interest by Alvis Suite (Ba and Bossy 2016) and then passed to seven annotators to revise. Since we have decided not to deal with microorganisms in the current work, the annotations of bacterial taxon names are not useful to us. Due to the taxon differences, the annotations of habitats are also different. Bacteria Biotope consists of bacterial habitats, e.g. diseases, symptoms, experimental materials and methods, which are out of the scope of this work.

ACE 2005 (Walker et al. 2006) can be considered as benchmark data for several natural language processing tasks, e.g. named entity recognition, relation extraction and coreference resolution, in the general domain. ACE 2005 consists of 599 documents extracted from six different genres: broadcast news, broadcast conversations, newswire, weblog, usenet and conversational telephone speech. The corpus was annotated with seven types of entities. i.e. persons, organisations, geographical/social/political entities (GPE), locations, facilities, vehicle and weapons, each of which was further divided into subtypes. Amongst these types, person and GPE are the only entity types that partially match our requirements. However, the guidelines show that ACE person entities are too general for our work. For species occurrence records, only specific person names are relevant, rather than other general instances such as "the butcher", "dad", "he", "the family", "the house painters" etc. Regarding GPE entities, they are too general and their scope is too narrow for our scenario. In order to provide detailed information about species occurrence, geographical coordinates should be identified as well as geographical names. However, coordinates are not available in ACE 2005.

The same situation applies to both CoNLL 2003 (Kim Sang and Meulder 2003) and MUC-7 (Chinchor 2001), which both include person and location annotations that do not match our needs. However, annotations of temporal expressions in MUC-7 are more closely aligned with the types of temporal expressions that we annotate, except for those of times, e.g. "9 o'clock" and "8 A.M", which are not of interest to us.

In contrast to previous work in the biodiversity domain, which has focused only on taxon names or microogranisms and their habitats, or other work in the general domain, whose annotated entity types only partially overlap with the types of information that are of interest to us, the work described in this article has produced a corpus that is especially designed for the biodiversity domain, including documents relevant to this domain. The corpus has been manually annotated with domain-specific entities belonging to five different semantic categories. These categories were chosen with the specific target of detecting species occurrences from literature.

## Materials and methods

In this section, we describe in detail how we constructed the gold standard corpus. We present our method of selecting the data, the annotation guidelines and the annotation process.

### Data selection

The source of data for our corpus is the Biodiversity Heritage Library (BHL)---an open access library that has digitised millions of pages of legacy literature on biodiversity spanning over 200 years (Gwinn and Rinaldo 2009). For this work, we focused on the requirements of the COPIOUS project, which is concerned with extracting information about species distribution in the Philippines and reproductive patterns of Philippine Dipterocarps. To obtain documents relating to Philippine species distribution, we programmatically downloaded documents from the English BHL pages that mentioned either "Philippine" or "Philippines" and pages of books or volumes whose titles mentioned either "Philippine" or "Philippines". Reproductive patterns in tropical forest trees (Dipterocarps in this work) are associated with the timing of seasonal events such as budburst, flowering, fruiting and sterility. To select documents relevant to the reproduction of Dipterocarps, we searched pages that contained any of the six genera of the Dipterocarpaceae family, namely, *Anisoptera*, *Dipterocarpus*, *Hopea*, *Parashorea*, *Shorea* and *Vatica*, together with the word "flower" or "fruit". The downloading and searching programmes were implemented by using BHL's publicly available application programming interface (API)*^5^. The API provides functions for retrieving the OCR text of each document according to specific conditions, e.g. keywords or the document's language. As a result, we obtained more than 169K BHL pages; 668 of them were randomly selected as the basis of our gold-standard corpus, which would be annotated by experts with biodiversity named entities.

### Annotation guidelines

As mentioned above, we annotated five categories of entities in our corpus, i.e. taxon names, geographical locations, habitats, temporal expressions and persons. Details of each category are described in the following subsections. It should be noted that, in the examples provided, annotations in square brackets should be annotated while the underlined terms should not be annotated.

#### Taxon

Taxon entities are expressions that pertain to members of the taxonomic ranks, e.g. species, genus, family etc. Specifically, we annotated current and historical scientific names (e.g. [[*E. salmonis*] Müller, 1784]]; [[*Salvelinus alpinus*] (L.)]). For scientific names that include authorship information, two overlapping entities (with/without the authorship) are annotated as shown in the examples. In this category, vernacular names of species (e.g. [flying fox], [insectivorous bats]) are also marked up. However, vernacular names of taxonomic classes for general species, i.e. general names such as fish, birds, mammals, reptiles, amphibians, animals and plants, were not tagged as taxon entities. For example, "birds" in "A few birds seem to range widely from ..." and "amphibian" in "… a list of amphibian known from South Gigante Island" are excluded from annotation. In contrast to the Bacteria Biotope corpus (Delėger et al. 2016), microorganisms are not within the scope of our current work and hence all microorganism taxon names are excluded from annotation. Modifiers derived from organism names (e.g. a noticable porncine smell) and descriptive references (e.g. [*H. lasiocarpus*], the large and bushy perennial herb with sprawling stems reaching up to two metres long) were neither tagged as nor included with Taxon entities. Modifiers which are not part of the name (e.g. tuberous-rooted [begonias]) and characters occurring within the same token as the name but which do not form a part of it (e.g. [corn]-based products) were also excluded from annotation.

#### Geographical location

Mentions of geographical locations, i.e. any identifiable point or area in the planet, ranging from continents, major bodies of water (e.g. oceans, rivers, lakes), named landforms, countries, states, cities and towns, were marked up as geographical location entities. These types of mentions do not only include Philippine geographical locations but also worldwide locations (outside of the Philippines). Similarly to several corpora annotated with geographical entities, e.g. MUC-7 (Chinchor 2001), CoNLL 2003 (Kim Sang and Meulder 2003), ACE 2005 (Walker et al. 2006) and Bacteria Biotope (Delėger et al. 2016), we labelled instances of geographical location proper names (e.g. [Steward Island], [East coast of Mindoro], [Balayan Bay]) and their abbreviations, except when used in a political context or when occurring in adjectival form (e.g. the Philippine Government). For the purpose of mapping species occurrence, we additionally annotated geographical coordinates (e.g. [N. 36^o^ E], 7.2 miles ([13^o^ 36' 11" N.], in [lat. 35° 21’ S], [long. 175° 40].) Informative modifiers, i.e. those which indicate a specific region of a location, e.g. "southern" in the text "[southern Philippines]" were included in the span tagged as geographical locations. It should be noted that coordinating words are excluded from entity spans. When entities are coordinated by such words, annotators were asked to create discontinuous entities. For example, a phrase such as "North or South Africa" should be annotated as a discontinuous entity, i.e. [North Africa] and a continuous entity, i.e. [South Africa], excluding the word "or" from entity spans.

#### Habitat

Habitat entities are mentions of environments in which organisms live. These are textual expressions describing natural environments, e.g. [Lowland forest], [coconut groves] and [banana plantations] and places where ectoparasites or epiphytes are residing, e.g. "… parasitic on [Achillea holosericea]". It should be noted that informative modifiers, i.e. those which provide information in terms of composition, altitude or weather conditions should be included in text spans, e.g. [subalpine calcareous pastures] or [rocky slopes]. Since microorganisms are excluded from the current annotation effort, their habitats, such as diseases, symptoms, experimental materials and methods, are excluded too. Other exclusions from annotations of habitats are (1) habitat attributes, i.e. altitude, depth, elevation or area, e.g. "In the [mossy forest], altitude about" and "... [second-growth forests] at elevations from ..."; (2) habitat attribute values, i.e. descriptive references containing numerical values to indicate habitat attributes, e.g. 12-29 fathoms or 520 metres. We also excluded modifiers that convey information within the context of a geographic location but not on their own, e.g. the western [slopes] and adverbs or prepositions that precede the habitat, e.g. under [logs] or [rocks]. Similarly to Geographical entities, each item within enumerations of habitat descriptions was tagged separately, i.e. coordinating words and characters, e.g. *and*, *or*, and commas were excluded from the annotation.

#### Person

We labelled proper nouns pertaining to person names, including generational suffixes (e.g. Jr and Jnr), used in the context of an occurrence or a historical account (e.g. "In 1905, [Tattersall] follows [Milne Edwards] in..."). Person names in citations that convey observations related to a species were marked up, e.g. "In the East China Sea, [Koto] et al. (1959) report that sailfish migrate northward...". However, we did not label them if they were not related to any observations, e.g. "These three genera included the main component species ... (Inoue & Hamid, 1994; LaFrankie et al., 1995)". Names of persons that appear as parts of taxon names (e.g. *Scolopsis bulanensis* Evermann & Seale) were not tagged. Titles (e.g. Dr. [Waring] recommends ...) and characters which are not part of the name but appear in the same token (e.g. Dr. [Johnston]'s findings) were also excluded from the annotation span. Unlike MUC-7 (Chinchor 2001), CoNLL 2003 (Kim Sang and Meulder 2003) and ACE 2005 (Walker et al. 2006), general references to people, such as "the researcher", "he", "they", "the family" and "the farmers" were not labelled.

#### Temporal expression

We annotated spans of text pertaining to points in time as temporal expressions. These expressions can be any mention of a specific date (e.g. [10 June 2013]), month or year (e.g. from [March] to [November]), decade (e.g. in the [1920s]), a regular occurrence, e.g. seasons and geochronological ages (e.g. during the [late Pleistoncene]). In contrast to temporal expressions in MUC-7, we did not mark up mentions pertaining to time-of-the-day information, e.g. "Specimens were found between 19:40 and 20:10". Similarly to Person entities, if temporal expressions in citations conveyed species observations, we annotated them. However, if they did not convey such observations, we did not annotate them, e.g. "In the East China Sea, Koto et al. ([1959]) report that sailfish migrate northward...". Expressions used as part of a taxonomic name's authority (e.g. *Emesopsis infenestra* Tatarnic, Wall & Cassis, 2011) were not tagged as temporal expression. Characters and coordinating words used to indicate a range (e.g. words "from" and "to" in the previsous example) were also excluded from the tagged span of text.

The detailed guidelines, with further instructions and more examples, are provided in Suppl. material 1.

### Annotation Process

We firstly recruited two annotators with expertise in biology: one a master student and the other a graduate with a BSc. We then conducted a two-stage annotation process. In the first stage, we randomly selected 200 documents for double annotation by the two annotators. During this stage, the annotators were encouraged to provide us with feedback or comments to improve the annotation guidelines. We iteratively revised the guidelines and the annotations until we obtained an acceptable inter-annotator agreement between the two annotators. In the second stage, each annotator was assigned a separate portion of the 468 remaining documents to annotate.

**Figure 1.**
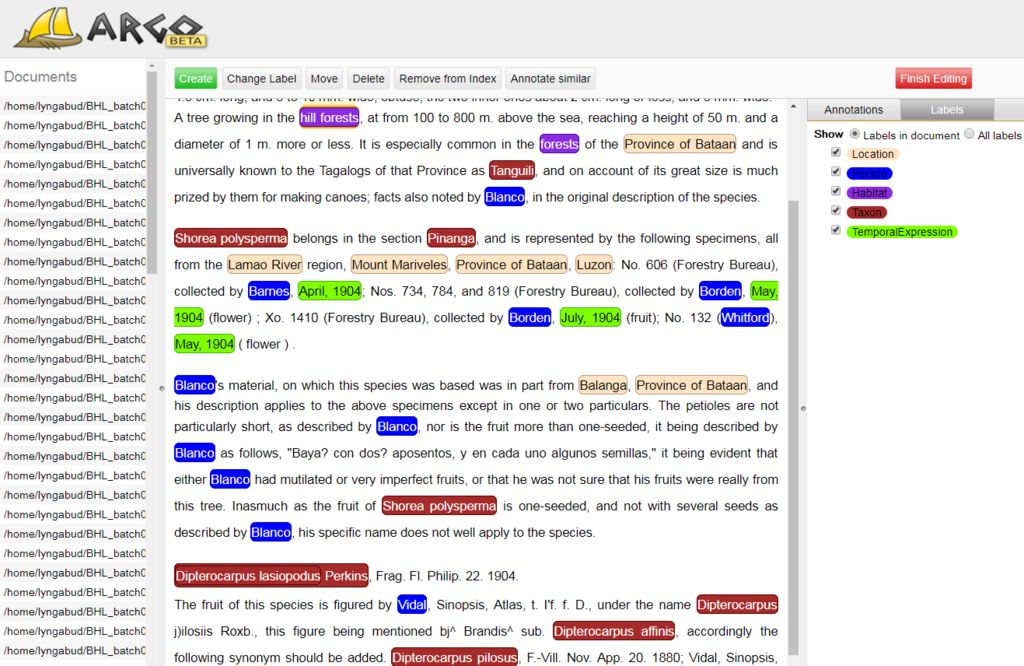
Argo’s Manual Annotation Editor to support annotators. Each entity category is represented using a different colour.

In order to support the annotators, we utilised Argo, a workbench for building text mining solutions (Rak et al. 2012). Argo is a web application that does not require any complicated platform-dependent installation procedures*^6^. It has its own library of text processing components and a file store to hold document collections. We made use of the Argo’s Manual Annotation Editor to create and revise annotations directly in text. Fig. 1 shows the graphical user interface of the Editor. It is a convenient and straightforward tool for domain experts to mark-up documents according to a user-specified annotation schema. The Editor allows annotators to change annotation labels and to move or delete annotations easily. An annotator can also quickly tag similar entities by using a function called ''Annotate similar''.

## Results

In this section, we present details of the COPIOUS corpus and the results of NER experiments applied to the corpus. We conducted two different NER experiments. Firstly, we trained NER tools using CRF (Lafferty et al. 2001) and BiLSTM (Espinosa et al. 2016) models. Secondly, we compared the outputs of the resulting NER tools with those of the Global Names recognition and discovery tool (GNRD)*^7^ and the SPECIES tagger (Pafilis et al. 2013) on the task of detecting species names.

### The gold standard

During the first stage of the annotation process, we calculated the inter-annotator agreement (IAA) between the two annotators using F-scores. We applied 'strict' matching criteria, which means that the two annotators were considered to agree on a named entity only if they tagged *exactly* the same span *and* assigned the same entity category. Table 2 presents the IAA of the two annotators over 200 doubly-annotated BHL pages.

The level of agreement between the two annotators was high for most of entity types, except for Habitat. Aside from usual human errors, e.g. confusions between Geographical Location and Habitat entities, the disagreements between the two annotators with Habitat entities were often due to the specificity of the expressions in this category. Specifically, one annotator tagged more general habitat terms while the other tagged longer and more descriptive terms. Examples include "extensive [forests]" vs "[extensive forests]" and "margins of [primitive forests]" vs "[margins of primitive forests]". Another reason is the inclusion of adverbs or prepositions in between two general habitat terms that may pertain to a more specific habitat description. In such cases, one annotator tended to exclude the adverbs or prepositions and tagged two separate habitat terms, while the other annotator included the prepositions and tagged as only a single habitat term. For instance, "[primary forest] on [hilly ground]" vs "[primary forest on hilly ground]" and "[damp ravines] at [low altitudes]" vs "[damp ravines at low altitudes]". Although it was mentioned in the guidelines that coordinating words should be excluded, one of the annotators sometimes made mistakes, e.g. by annotating "[hilly or steep localities]" instead of "[hilly] or [steep localities]".

After the double annotation stage, we asked each annotator to label their own individual sets of documents. As a result, our gold standard consists of 668 documents. The numbers of sentences, words and entities of the corpus are presented in Table 3. Amongst the five entity categories, Taxon and Geographical Location have the most instances, while Habitat is the sparsest entity type.

The gold standard is publicly available at: http://nactem.ac.uk/copious/copious_published.zip.

### Named entity recognisers

We randomly divided the annotated corpus into three different sets: (1) the training set with 80% of the data (543 documents), (2) the development set with 10% (67 documents) and (3) the test set with the remaining 10% (67 documents). This division is provided in Suppl. material 2. The distribution of entities on each subset is roughly representative for that of the whole corpus, as illustrated in Table 4.

We trained both CRF and BiLSTM models by using the training set, tuned the models using the development set and evaluated their performance using the test set. To train the CRF model, we used NERSuite (Cho et al. 2013) with basic features, i.e. word base form, part-of-speech tag and chunk tag of each token, obtained using the GENIA tagger (Tsuruoka et al. 2005). Meanwhile, for the BiLSTM model, we used pre-calculated word embbedings, trained on the English subset of BHL (Nguyen et al. 2017) as input. The results of the two models are reported in Table 5. Similarly to the IAA calculation, we also used strict matching to compute these scores.

Amongst the five categories, CRF performed the worst on Habitat entities, with an F-score of 52.11%. This is expected, as the number of Habitat entities is the lowest amongst all the categories (as shown in Table 3). Additionally, the fact that Habitat also exhibited the lowest IAA of all categories (in Table 2) shows that this category is more difficult than the others to determine. If humans struggle to annote the correct spans, then it follows that the computer will also have problems in predicting them. Issues that were revealed from the IAA analysis above cascaded to the NER results. We also noticed that Habitat mentions that start with uppercase letter, either due to the word being a proper noun or as a beginning of a sentence, e.g. Antarctic marine, Malayan forest and Mouths of rivers, were often missed by the model. It should be noted that the NER performance for Habtiat entities is low, not only with this gold standard, but also with Bacteria Biotope, in which habitat annotations were the most numerous (1,921 entities compared to 966 bacteria entities). Lavergne et al. (2015), Delėger et al. (2016) and Mehryary et al. 2017 reported that their models trained on Bacteria Biotope performed worse on Habitat entities than on the other types of entities. These results reinforce our previous suggestion that, in general, habitat entities described in text are complex phenomena, which are probematic both to annotate and to detect automatically.

The second lowest F-score for the CRF model is 54.15% for Person entities. Identifying Person names was challenging for a number of reasons. Firstly, they can sometimes be a part of a Taxon name, leading to the confusion between Person and Taxon entities. We observed that Person entities with abbreviations, i.e. containing comma or full stop within the text, e.g. Alonzo, S., Apostolaki, P,E., were sometimes predicted as part of a Taxon name. Determining whether Person name forms part of a citation could also be confusing. The model also failed to recognise some instances of Person names that are followed by a year inside a pair of parentheses that pertain to actual observation, e.g. "[Voss] (1953) believe that there be a population of sailfish present". Furthermore, Person names that were spelled in all uppercase were not identified by the model. The general performance of the CRF model over all 5 entity types was acceptable, with an F-score of 71.53%.

Regarding the BiLSTM approach, the performance on Person entities was surprisingly low. Similarly to the CRF model, the BiLSTM model often tagged person names in citations and species names, even though these mentions should be excluded. For example, BiLSTM labelled "Schepman'' in "... a foreign journal (Schepman, 1907)'' as a Person entity, which is not correct according to our annotation scheme. Another reason for the low performance is that the model sometimes confused Person and Geographical Location entities. For instance, "Charles Glass" in "... have been received from Charles Glass of Santa Barbara ...'' should be a Person name, while the model included the whole name in a Geographical Location entity as "Charles Glass of Santa Barbara''. In contrast, "Ringim Mukr'' in "... Ringim Mukr, 2500 ft., flowers bright pink ...'' should be a Geographical Location entity, but the BiLSTM tagged it as a Person.

Although the BiLSTM model obtained higher scores than those achieved by the CRF model for the majority of categories, the overall performance of the two models was not significantly different. It can be seen that BiLTSM had wider coverage, i.e. higher recall, for all categories, but in some cases, e.g. Person and Temporal Expression, it was less precise than the CRF model. This can be explained by the fact that the BiLSTM only used word vectors as input features, while the CRF model used advanced features, namely POS and chunk tags. However, the fact that both types of models obtained good results serves to demostrate that our corpus has potentially wide utility for developing NER tools for biodiversity.

### External comparisons

To the best of our knowledge, there is no available tool that can automatically detect all of the above-mentioned categories of named entities in biodiversity texts. Rather, the only other relevant tools that are currently available are those that can detect taxon names (Akella et al. 2012, Boyle et al. 2013, Pafilis et al. 2013, Pyle 2016, Rees 2014). We therefore selected two of these external tools, i.e. GNRD (Pyle 2016) and the SPECIES tagger (Pafilis et al. 2013) as points of comparison for the taxon recognition performance of the NER tool trained on our corpus (the BiLSTM model, since this obtained better overall performance than the CRF model). GNRD is a combination of TaxonFinder*^8^ and NetiNeti (Akella et al. 2012) that focuses on finding scientific names. Meanwhile, the SPECIES tagger and our NER can detect both scientific and vernacular names. To conduct the comparisons, we applied GNRD and the SPECIES tagger to our test set.

The results reported in Table 6 show that GNRD can obtain good performance in recognising species names in text. Its precision is competitive to that of our NER. However, GNRD only detects scientific names and ignores common names, which is a reason for its low recall. Another limitation of GNRD is that it overlooks species names that include authority fields, i.e. the name of the first person to publish it and the year that it was coined, e.g. [*Murina cyclotis* Dobson, 1872].

Since the SPECIES tagger detected species names based on the NCBI Taxonomy (Pafilis et al. 2013), it is reasonable that the tagger obtained the highest precision but the lowest recall. The tool often failed to capture species names that are not included in NCBI Taxonomy, such as [*Cerithium torresi*], [*Cavallium urens*] and [molave tree]. Moreover, regarding scientific names, the tool could only identify names in the format of binomial nomenclature, hence ignoring genus names (e.g. [*Platymantis*]), subspecies names (e.g. [D. turbinatus var. andamanicus]) and names with authority fields (similarly to GNRD).

In terms of F-scores, the model trained on our gold standard could attain better performance than both the GNRD and SPECIES tagger.

## Species occurrence extraction

Occurrence data and species distribution play an important role in monitoring as well as preserving the biodiversity (Chapman 2005, Soberón and Peterson 2009, Parr and Thessen 2018). Primary occurrence data are manually extracted from observational notes or from data inherent in museum and herbarium collections (Chapman 2005, Parr and Thessen 2018). However, such occurrences are also frequently mentioned in literature. With the availability of our gold standard corpus, we can develop a system that automatically discovers species occurrence records from literature.

**Figure 2.**
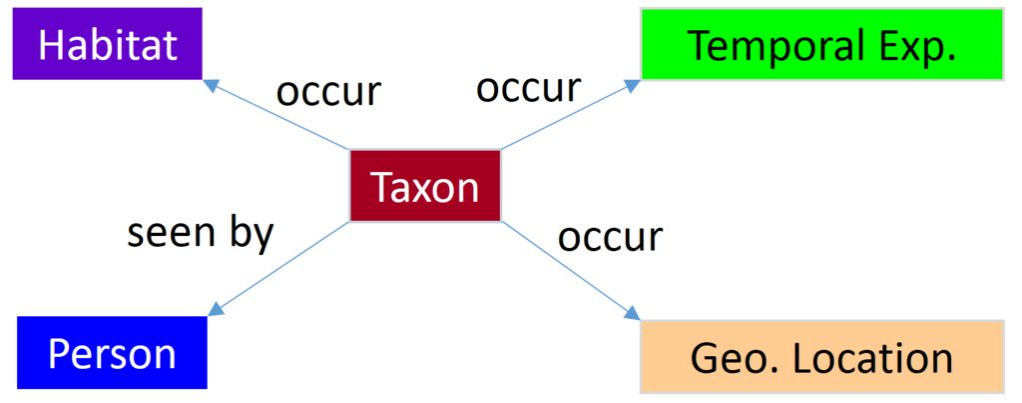
Schema of occurrence extraction.

To this end, we firstly consulted our domain experts to define a schema for relations between entities for species occurrence records. The schema specifically describes two types of relations: *occur* and *seen_by*. *Occur* relations pertain to occurrence records of species, i.e. Taxon, in specific Geographical Locations or Habitats or at a point of time, i.e. Temporal Expression. Meanwhile, *seen_by* relations denote observations of a specific Person on specific species. Consequently, we attempted to identify four binary relations between Taxon entities and the other types of entities, as shown in Fig. 2. We secondly designed a two-step system: (1) to recognise named entities in texts and (2) to extract relations between these entities. For the first step, we used our gold standard corpus to train the BiLSTM model for the five categories. For the second step, because there are no gold standard annotated relations available for the defined binary relations, this task must be approached by using an unsupervised method. We therefore employed PASMED—a pattern-based system that can identify any binary relations between entities within a single sentence (Nguyen et al. 2015).

**Figure 3.**
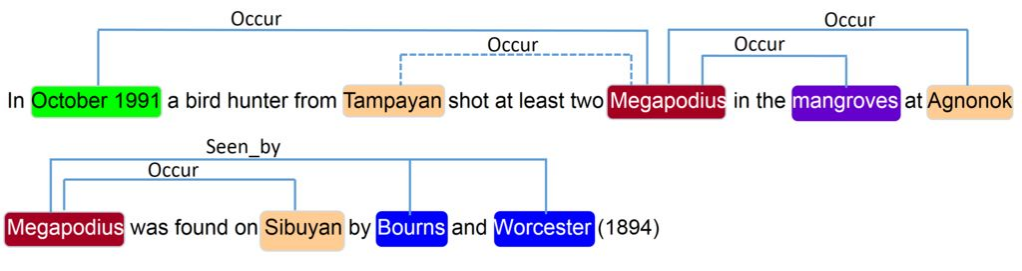
Examples of species occurrences automatically extracted by PASMED. A dashed line indicates an incorrect relation.

Since PASMED extracts relations based on predicate-argument structures, we firstly applied the Enju parser (Miyao and Tsujii 2008) to obtain these structures and then applied PASMED to the whole collection of 668 documents. As a result, we obtained 1,470 occurrences (i.e. *occur* relations) and 200 *seen_by* relations. Fig. 3 illustrates some examples of the extracted relations. As shown in this figure, the system predicted four *occur* relations in the first sentence, but one of them (the one with the dashed line) is not correct. Meanwhile, the second sentence conveys one *occur* and two *seen_by* relations, which were correctly identified.

Extracting species occurrences from text would be an initial step towards developing a semi-automatic system that can complement the primary data of species occurrences with those described in literature. A potential system would consist of three steps. The first step is to ask domain experts to verify the extracted species observations. The second step is to normalise taxon names, geographical locations and habitats. Finally, we can straightforwardly convert the normalised information into Darwin Core Standard (Wieczorek et al. 2012) to make them compliant with several primary data in biodiversity, such as GBIF, to support prediction of new distributions of species (Pearson et al. 2006) and understanding of species declines over time or over areas (Soberón and Peterson 2009). We however leave it as future work.

## Conclusions

In this paper, we have described the process of constructing the COPIOUS corpus, which is annotated with five entity categories relevant to the study of biodiversity: Taxon names, geographical locations, habitats, temporal expressions and persons. With 668 documents and 28,801 entities annotations, the corpus is sufficiently large for both training and evaluating text mining tools. Our experimental results have demonstrated that the corpus is useful for text mining biodiversity texts in terms of both NER and occurrence extraction.

As future work, we aim to improve the performance of the NER tools, especially for the most problematic categories of Habitat and Person and then to apply the NER to the whole collection of BHL English pages. This will allow us to produce another semantic layer for BHL documents, in addition to the current layer of annotated scientific names, which should pave the way for an advanced semantic search system over the BHL. Another long-term goal is to extract species occurrence data from the whole BHL collection using the two-step method of occurrence extraction. Although our gold standard was developed specifically for the use case of Philippine species, the corpus is general enough to be employed for the whole BHL. However, beyond the large amount of computation that will be required to do this, there is one further limitation in terms of scaling up the task: BHL documents contain a large number of misspelt words, which are caused by errors from OCR tools and such errors may adversely affect the NER performance. Accordingly, we are investigating the application of OCR correction tools, such as, Thompson et al. 2015 and Mei et al. 2018, which were designed to correct historical text.

## Supplementary Material

Supplementary material 1 Named Entity Annotation Guidelines Data type: Annotation guidelines
Brief description: A .pdf file presents our guidelines to mark up five categories of entities. The guidelines provide specific instructions to annotators about the annotation scope and the annotation span of each category. Examples are used to demonstrate these instructions. The guidelines also describe some exceptions that the annotators must follow during their annotation process.
File: oo_252228.pdf Nhung T.H. Nguyen, Roselyn Gabud, and Sophia Ananiadou

Supplementary material 2 Division for training named entity recognisers Data type: Manually annotated data
Brief description: A compressed file contains three divided subsets: 80% for training, 10% for development and 10% for testing, used in our named entity recognition experiments.
File: oo_254879.zip Nhung T.H. Nguyen, Rosalyn Gabud, Sophia Ananiadou

## Figures and Tables

**Table 1.** Statistics of Linnaeus and S800 corpora for species names.

**Corpus**	**Document Type**	**Num. of Documents**	**Num. of Sentences**	**Num. of Words**	**Num. of Mentions**
Linnaeus	PMC full paper	100	17.580	502,507	4,259
S800	PubMed abstract	800	8.064	201,981	3,708

**Table 2.** Inter-annotator agreement on different named categories over 200 doubly-annotated documents. The categories are arranged in descending order of agreement.

**Category**	**Precision**	**Recall**	**F-score**
Geographical Location	94.32	94.89	94.60
Person	88.93	91.76	90.33
Temporal Expression	86.59	87.25	86.92
Taxon	81.09	83.87	82.45
Habitat	45.85	48.36	47.07
**Overall**	82.09	81.62	81.86

**Table 3.** Statistics of the gold standard corpus. The categories are arranged in descending order of the instance number

Number of documents		668
Number of sentences		26,277
Number of words		33,475
Number of entities	Taxon	12,227
	Geographical Location	9,921
	Person	2,889
	Temporal Expression	2,210
	Habitat	1,554

**Table 4.** The distribution of entities in training, development and test sets.

**Category**	**Train**	**Dev**	**Test**
Taxon	9,357	1,548	1,322
Geographical Location	8,121	992	878
Person	2,479	180	230
Temporal Expression	1,800	157	253
Habitat	1,308	91	115

**Table 5.** Performance of CRF and BiLSTM on the testing set. The categories are arranged in descending order of F-score for each type of model.

**Model**	**Category**	**Precision**	**Recall**	**F-score**
CRF	Geographical Location	82.35	83.49	82.92
	Taxon	75.27	62.40	68.23
	Temporal Expression	77.19	52.17	62.26
	Person	72.82	43.10	54.15
	Habitat	63.55	44.16	52.11
	**Overall**	77.67	66.29	71.53
Bi-LSTM	Geographical Location	85.05	85.63	85.34
	Taxon	77.42	69.67	73.34
	Habitat	64.10	64.94	64.52
	Temporal Expression	70.67	54.36	61.45
	Person	58.92	48.44	53.17
	**Overall**	77.49	71.89	74.58

**Table 6.** Performance of different NER tools on Taxon entities in the COPD corpus test set. In this table, we report the best performance for taxon names by the BiLSTM model.

**Tool**	**Precision**	**Recall**	**F-score**
Our NER (BiLSTM)	77.42	69.67	73.34
GNRD	77.61	54.02	63.70
SPECIES Tagger	86.79	4.51	8.57

## Footnotes

*1 Biodiversity Heritage Library. https://www.biodiversitylibrary.org

*2 Conserving Philippine biodiversity by understanding big data (COPIOUS). NaCTeM. http://nactem.ac.uk/copious

*3 Global Biodiversity Information Facility. https://gbif.org

*4 NCBI Taxonomy Database. https://www.ncbi.nlm.nih.gov/taxonomy

*5 Biodiversity Heritage Library API v2 Documentation. http://www.biodiversitylibrary.org/api2/docs/docs.html

*6 ARGO: A Web-based Text Mining Workbench by he National Centre for Text Mining. http://argo.nactem.ac.uk

*7 Global Names Architecture. Global Names Recognition and Discovery. http://gnrd.globalnames.org

*8 Taxon Finder. http://taxonfinder.org

## Hosting institution

The National Centre for Text Mining, University of Manchester, Manchester, United Kingdom
