# Supplementary material for: COPIOUS: A gold standard corpus of named entities towards extracting species occurrence from biodiversity literature
Source: Biodivers Data J. 2019 Jan 22;(7):e29626. doi: 10.3897/BDJ.7.e29626 (PMC6351503; doi:10.3897/BDJ.7.e29626)
Supplement: Supplementary material 1 — Named Entity Annotation Guidelines [file bdj-07-e29626-s001.pdf]

COPIOUS - Named Entity Annotation Guidelines

A. Introduction 2

B. Detailed guidelines [1] 3

1. Taxon 3

1.1 Annotation scope 3

1.2 Annotation span 4

2. Geographic location 4

2.1 Annotation scope 4

2.2 Annotation span 5

3. Habitat 6

3.1 Annotation scope 6

3.2 Annotation span 6

4. Temporal expression 7

4.1 Annotation scope 7

4.2 Annotation span 8

5. Person 8

5.1 Annotation scope 8

5.2 Annotation span 9

C. An example of full annotations 9

D. External resources 9

E. Using brat to annotate .... **Error! Bookmark not defined.**

References 10

A. Introduction

In this project, we focus on extracting information from documents related to the Philippine biodiversity. Specifically, we target two main case studies (1) analysing species distribution and (2) detecting potential medicinal applications of natural products derived from Philippine species. In order to perform such case studies, one of the most important resources that we need is a labelled corpus of biodiversity entities, including taxon, geographic location, habitat, temporal expression, and person.

Henceforth, we denote that all expressions in a sentence that belong to the annotation category under discussion are enclosed in [square brackets]; expressions that could be considered as candidates for annotation, but which should actually not be annotated are enclosed in square brackets with a [~~strikethrough~~].

The task of an annotator is to strictly follow our detailed guidelines, described in the next section, to label the entities of interest within text. Moreover, the annotator should note some general rules [2] as follows, during the annotating process.

- **Do not tag erroneous words**. Most of texts have undergone optical character recognition (OCR) and thus contain a significant amount of typos. For such erroneous words, please skip them.
- **Do not tag unclear cases**. If the annotator is not sure about a given mention, even after consulting some external sources, the corresponding mention should remain unlabelled.
- **Do not tag pronouns or mentions that are used to refer to a specific entity**. An annotated entity should be a specific expression in its context and should not be a pronoun or a phrase that used to refer to a specific entity.

> [Chenopodium ambrosioides] is a branched herb nearly a meter in height ... ~~[This species]~~ is widely distributed in the Philippines, both cultivated and wild.
> Genus [CAESALPINIA] [[CAESALPINIA SAPPAN] L. SIBUKAU].
> A description of [~~this species~~] and [~~its~~] local names are given in the section on dyes.
> [Acetyl methyl salicylate]. — [~~This compound~~] was produced in a manner analogous to that employed by Freer …

- **Do not tag general words/phrases.** Please tag entities that convey a specific object, i.e., a scientific name or vernacular name of a species, a specific habitat, a specific compound, etc. rather than general groups or classifications.

> A few [~~birds~~] seem to range widely from the [~~lowlands~~] and up through the [~~mountains~~] …
> … occurring in open [~~places~~].
> [~~Protein~~] split products in relation to immunity and [~~disease~~] …
> … as feed for broodstock oysters provide greater variation of [~~fatty acid~~] than from one species of [Algae].
> … a list of [~~amphibian~~] known from [South Gigante Island] …

- **Note sentences' boundary**. Since sentences in texts are segmented automatically, the accuracy is not 100%. In the case that an entity is separated through two sentences, please skip it as the following example.

Sentence 1	A new frog of the genus **Cornufer**
Sentence 2	**(Ranidae)** with notes on other amphibians known …

- **Be careful with ambiguous expressions**. In case an expression can be classified into more than one category, the annotator must carefully analyse its context to decide the most appropriate category.

- **Use external resources**. In case an annotator is not sure if a mention, i.e., text span of single or multiple words, corresponds to a category or (s)he does not know what kind of mention it is, (s)he may consult external knowledge resources: Catalogue of Life, Encyclopedia of Life, Environment Ontology… A list of external resources is given in Section C.

B. Detailed guidelines [1]

1. Taxon

Taxon entities are expressions which pertain to members of any of the taxonomic ranks.

1.1 Annotation scope

✔Include:

- Any expressions that can be considered as a taxonomic name, both current and historical names

> Samples of [Echinorhynchus 'bothniensis'] were obtained from the following hosts: [[Salvelinus alpinus] (L.)], [[Coregonus lavaretus] (L.)] and [[Platichthys flesus] (L.)].

- Abbreviations of taxonomic names

> These [acanthocephalans] were collected by Shostak et al. (1986) during their extensive survey of morphological variability in [E. gadi], [E. leidyi] and [[E. salmonis] Müller, 1784] from northern Canada.

- Vernacular or common names of species

> The common island [flying fox] occurs from Thailand to Australia …
> The most abundant species of [fiddler-crab] along the esteros near Manila has not been described …

- Biotypes of species (both full names and abbreviations) that have been named in text

> The two biotypes of [Chromaena odorata] are the [Asian/West African biotype] ([AWAB]) and [southern African biotype] ([SAB]). [AWAB] originated from [Trinidad] and [Tobago] …

✘Exclude:

- General groups of species, e.g., birds, plants, mammals, ants, crocodiles, etc.

> Enumeration of Philippine [~~Plants~~]
> 45 [~~mammal~~] skins and skulls
> BIBLIOGRAPHY OF [~~FISHES~~]

- Modifiers derived from organism names,

> There is a noticeable [~~porcine~~] smell around the market.

- Descriptive references.

> [H. lasiocarpus], [~~the large, bushy perennial herb with sprawling stems reaching up to two meters long~~], is native to much of the southeastern United States.

1.2 Annotation span

Only the minimal span of text signifying the taxonomic entity should be annotated and nothing else.

✔Include:

- Authority names and nomenclatural acts,

Lectotype of [[Solanum jasminoides] **Paxt.**] reproduced with permission of the Natural History Museum Botany Library.

- In cases where the taxonomic name includes information pertaining to authority, the enclosed Latin name (i.e., genus and species) should also be marked up, as a separate annotation. In the example below, two annotations must be created: one for the whole span and another for just the Latin name.

This paper summarises our findings on [[Emesopsis infenestra] **Tatarnic, Wall & Cassis, 2011**].

✘Exclude:

- Modifiers which are not part of the name,

A description of how [~~tuberous-rooted~~] [begonias] was provided by Harrison et al.

- Characters appearing in the same token as the name but are not part thereof.

There is ongoing debate on the danger of [corn][~~-based~~] products.

2. Geographic location

2.1 Annotation scope

Mentions of geographic locations, i.e., any identifiable point or area in the planet, ranging from continents, major bodies of water (e.g., oceans, rivers, lakes), named landforms, countries, states, cities and towns, are marked up as geographic location entities. It should be noted that this type of mentions does not only include Philippine geographic locations but also world-wide locations (outside of the Philippines).

✔Include:

- Instances of proper names and their abbreviations, except when used in the context as a political entity.

Distribution: Endemic to the [Philippines]; [Luzon], [Palawan], [Basilan], [Culion], [Balabac], [Mindoro], [Mindanao], [Leyte], [Busuanga], [Panay].<br>U. ovaujolia Blunie . c. 27. — [Luzon], [Prov. Tayabas]; [Mindanao], [Misamis] ;<br>A tree which occurs in remaining patches of kerangas, vegetation in [Brunei] and [Sarawak].<br>The first group is composed of the bats, four of which are widely distributed in [Southeast Asia].<br>Habitat – [Steward Island].

- Geographic coordinates

Collected from D5122, [Malabrigo Light], [East coast of [Mindoro]], [N. 46° W.], 2060 miles ([13° 21' 30" N.]; [120° 30' 33" E.]) 220 fathoms, green mud bottom. D5114, [Sombrero Island], [Balayan Bay], [N. 36° E], 7.2 miles ([13° 36' 11" N.]; [120° 45' 26" E.]) 340 fathoms, fine sand bottom.

> The only species of this genus, Sclerisis pulchella, Studer, was found at a depth of 597 fathoms, in [lat. 35° 21’ S], [long. 175° 40].

✘ Exclude:

- Expressions which refer to locations but used in a political context or used as adjective roles,

> … the [~~Philippine~~] Government provided regulations for the grading and labelling to be done …
> [Mount Malindang] has been the least explored of the five [~~Philippine~~] mountain masses …

- Ambiguous references

> Specimens were gathered from the [~~mountains of~~ [northern Greece]].

2.2 Annotation span

✔Include:

- Text spans which appear as fragments of coordinated names

> The species was found in both [Lakes Tahoe] and [**Reno**].
> → The above annotations mean [Lake Tahoe] and [Lake Reno]
> It is a perennial shrub native to tropical [Central] and [**South America**].
> → The above annotations mean [Central America] and [South America]

- Informative modifiers, i.e., those which indicate a specific region of a location

> They carried out their field work in [**southern** France].

- Common words which appear as part of the name, i.e., the proper noun.

> Homalanthus populneus forming second-growth forest in [Bataan **Province**] … [Luzon], [**Province** of Pampanga], [Mount Arayat], Merrill 5026

- When several locations are grouped, individual locations should be annotated.

> .. [Tablas]–[romblon]–[sibuyan] group …

✘ Exclude:

- Coordinators when they are used to combine different location names

> The species was found in both [Lakes Tahoe] [~~and~~] [Reno]. (Please add a fragment of Lakes and Reno.)

- In cases where a continuous span of text consists of mentions which themselves individually pertain to geographic locations, only the individual constituents should be annotated, even if as a whole they help specify only one location.

> Helicostyla mindoroensis (Chrysallis) 1933, Pap. Mich. Acad. Sci. Arts Lett, 17: 549, pi. 58, fig. 1 (near [Calavite Mt], [Binuangan], [Paluan], [Mindoro], [Philippines]);

- Common words which do not appear as part of the name.

> [Lamao] ~~river~~, 135 meters, on damp rock and wood by stream …
> This province surrounds the [South Pole], and comprises the region corresponding to the [Arctic] ~~province~~ …

3. Habitat

3.1 Annotation scope

All mentions of habitats, i.e., environments in which organisms live, should also be annotated.

✔Include:

- Common nouns that indicate environments in which organisms dwell

  Habitat — [Lowland forest], [coconut groves], and [banana plantations].
  Habitat: [forest], [second growth and scattered trees] in open country.
  Habitat: [Artificial containers] and [rock pools]. [Fresh or saline water] near [seacoast].

- Taxon or species names[1] if they are places where ectoparasites or epiphytes are residing.

  … this species was found in [arboreal ferns] ranging from about 2-24m …
  … parasitic on [Achillea holosericea].
  Habitat — Philippines. On [cocoanut palm].

- Anatomies if they are places where ectoparasites or epiphytes are residing.

  … specimens are from [leaf axils of gabi plants] …
  … among [leaves and duff of the forest floor] …

✘Exclude:

- Names of geographic locations. They should be marked up using a different label (see above),

  Habitat — [~~Lahore~~], [~~India~~]

- Descriptive references containing numerical values to indicate altitude, depth or area.

  Habitat. — Philippine Islands. Depth— [~~12-29 fathoms~~].
  Habitat. — Near Timor. Depth. — [~~520 meters~~]

- Common nouns indicate habitats but play roles as modifiers

  … a ~~[desert]~~ crop

- Common nouns indicating general habitats (e.g., places, areas, regions, slopes) that are NOT modified by any habitat attributes.

  … over large ~~areas~~.
  … the fishermen work the entire ~~area~~ …
  … occurring in open ~~places~~ …

3.2 Annotation span

✔Include:

- Informative modifiers, i.e., those which provide information in terms of composition, altitude or weather conditions.

  On Leyte, specimens were netted in [**disturbed**] and [**old-growth lowland** forest] (create fragments to these entities when annotating)
  [**Dry**], [**subalpine calcareous** pastures] or [**rocky** slopes] (create fragments for "dry pastures" when annotating)

---

[1] It should be noted that if the taxon or species names occur in the same document but in their own context, i.e., describing their distribution or their characteristic rather than a parasitic site, they should be annotated as "Taxon" as normal. This rule will be applied similarly to anatomy entities mentioned below.

… [**agricultural**] and [**forested** areas] … (create fragments to these entities when annotating)
Habitat: [**cultivated** areas]
Habitat: Bohol. In [**shallow** water] and up to 10 fathoms.

✘ Exclude:

- Modifiers that convey information within the context of a geographic location but not on its own.

  [Rocky limestone slopes] at Lake Ohrid in F.Y.R. Macedonia, and the ~~western~~ [slopes] of Dry Mt, only a few metres from the locality of Centaurea soskae.

- Adverbs or prepositions precede the habitat.

  … ~~along~~ the [roads] …
  … ~~under~~ [logs] or [rocks] …
  … ~~in~~ the [shrub] …

4. Temporal expression

4.1 Annotation scope

Spans of text pertaining to points in time are annotated as temporal expressions.

✔ Include:

- Any mention of a specific date, month or year used in describing an occurrence, i.e., an account of a historical event,

  On [10 June 2013], I collected a single specimen of an emesine reduviid amongst long grass in a weedy overgrown wasteland area within the Tamaki Campus (East) of the University of Auckland.

- Expressions of decades

  … it was introduced from South America through the country's southern backdoor in the [1960's]
  … in [1920s] …

- Expressions which are part of a range of points in time,

  Conversely, the only two recent records of Microgaster deductor correspond to Canadian localities at 69–70°N, which had similarly been sampled in the [1940]–[1960] without finding any record of the species.

- Any temporal expression that provides information about a regular occurrence, e.g., seasons.

  The beetles were found active from [March] to [November] and it is possible that some species may be active during [winter] months, especially during mild [winters]

- Any temporal expression related to geochronological ages.

  … connected by land bridges to much larger islands during the [late Pleistoncene] …

- Expressions appearing as parts of a citation convey species observations.

  In the East China Sea, Koto et al. ([1959]) report that sailfish migrate northward …

✘ Exclude:

- Expressions used as part of a taxonomic name's authority,

> Emesopsis infenestra Tatarnic, Wall & Cassis, [~~2011~~] (Heteroptera: Reduviidae) is reported from New Zealand for the first time, based on a single specimen collected alive in the wild in Auckland in [June 2013].

- Expressions used as part of citations but do not convey any species observation,

> It is easily identified as Emesopsis infenestra from the original description (Tatarnic et al. [~~2011~~]).

- Mentions pertaining to time-of-the-day information

> Specimens were found between [~~19:40~~] and [~~20:10~~] on a small bush stem ca. 40 cm above the ground, approximately 3 m away from a rocky stream.

4.2 Annotation span

✔Include:

- The longest possible sequence of tokens pertaining to a point in time

> On trees in Cawsey Wood, [**January, 1801**], and at Gibside, Allansford, and Hamster ley, [1802], D. Behind Fenham, on Newcastle Town Moor, N., [1803].

- Modifying adjectives of geochronological ages, e.g., “late” “early” “middle” …

> … connected by land bridges to much larger islands during the [**early** Pleistoncene] …

✘Exclude:

- Words used to indicate a range.

> These two localities are 6–10° north of the Canadian collections from the [1950] [~~to~~] [1960].

5. Person

5.1 Annotation scope

Names of people should also be annotated.

✔Include:

- Proper nouns pertaining to person names, used in the context of an occurrence or a historical account.

> In 1905, [Tattersall] follows [Milne Edwards] in referring Noesa depressa to [Leach]. [Leach], however, never described this form, the earliest description having been given by [Say] in 1818, and the next to follow being that of [Milne Edwards] in 1840.

- Person names appearing as parts of a citation convey species observations.

> In the East China Sea, [Koto] et al. (1959) report that sailfish migrate northward …

✘Exclude:

- Person names appearing as parts of a taxonomic name,

> 164. Scolopsis bulanensis [~~Evermann~~] & [~~Seale~~], new species.

- Person names appear as parts of a citation but do not convey any species observations.

[~~Kamimura~~] 1968: 15 (as possible malaria vector, Japan); [~~Reisen~~] et al. 1972: 319 (distribution, Guam); [~~Zhang~~] et al. 1980:140 …

- Person names appearing in book/article/paper titles

Guide to Philippine flora and fauna: Amphibians and Reptiles. Univ. Philippines 101-195. [~~Alaca, A. C~~]. and [~~W. C. Brown~~], 1957

5.2 Annotation span

✔Include:

- Generational suffixes

The information was recorded by [John Dennis, **Jr.**] in 1976.

✘Exclude:

- Titles,

~~Dr.~~ [Waring] recommends the practice and ~~Dr.~~ [Van Soraeren] follows it in the application of water dressings, having substituted banana leaves for gutta-percha.

- Characters which are not part of the name but appear in the same token.

This was confirmed by Dr. [Johnston][~~'s~~] findings.

C. An example of full annotations

[Chromolaena odorata]$_{Taxon}$ (formerly known as [Eupatorium odoratum] $_{Taxon}$), also known as [Devil weed] $_{Taxon}$, [Christmas bush] $_{Taxon}$, [Common floss flower]$_{Taxon}$, or [Siam weed]$_{Taxon}$, is a [perennial shrub] $_{Taxon}$ that belongs to the [Asteraceae]$_{Taxon}$ family. It is also called [Hagonoy]$_{Taxon}$ in Tagalog, a common name shared by [Chromolaena odorata]$_{Taxon}$ and [Wedelia biflora]$_{Taxon}$ in the [Philippines]$_{Location}$. It is a [perennial shrub]$_{Taxon}$ native to tropical [Central] $_{Location}$ and [South America]$_{Location}$, and now common in range lands of humid tropics in [sub-Saharan Africa]$_{Location}$, [Asia]$_{Location}$ and [Oceania]$_{Location}$. The two biotypes of [Chromaena odorata]$_{Taxon}$ are the [Asian/West African biotype]$_{Taxon}$ ([AWAB]$_{Taxon}$) and [southern African biotype]$_{Taxon}$ ([SAB]$_{Taxon}$). [AWAB]$_{Taxon}$ originated from [Trinidad]$_{Location}$ and [Tobago]$_{Location}$, which can now be found in [West]$_{Location}$ and [Central sub-Saharan Africa]$_{Location}$, [Asia]$_{Location}$, [India]$_{Location}$ and [Oceania]$_{Location}$. On the other hand, [SAB] $_{Taxon}$ originated from [Jamaica]$_{Location}$ or [Cuba]$_{Location}$ and is now found only in [southern sub-Saharan African countries]$_{Location}$. [C. odorata]$_{Taxon}$ has become common in the [Philippines]$_{Location}$ since it was introduced from [South America]$_{Location}$ through the country's southern backdoor in the [1960's]$_{TemporalExpression}$.

D. External resources

Encyclopedia of Life http://eol.org/
Catalogue of Life http://www.catalogueoflife.org/col/search
Global Biodiversity Information Facility http://www.gbif.org/
World Registered of Marine Species http://www.marinespecies.org/
FishBase http://www.fishbase.org/
AmphibiaWeb http://amphibiaweb.org/
Environment Ontology
http://bioportal.bioontology.org/ontologies/ENVO/?p=classes&conceptid=root
ChemSpider http://www.chemspider.com/

Sigma Aldrich - Chemical Suppliers Catalogues http://www.sigmaaldrich.com/
Disease Ontology http://disease-ontology.org/

References

[1] Mining Biodiversity – Concept Annotation Guidelines. http://wiki.miningbiodiversity.org/doku.php?id=guidelines
[2] CHEMDNER data preparation and annotation manual. Version 2.0. 2013.
[3] http://brat.nlplab.org/introduction.html
